# Surgery of congenital breast asymmetry-which objective parameter influences the subjective satisfaction with long-term results

**DOI:** 10.1007/s00404-021-06392-1

**Published:** 2022-03-10

**Authors:** Vivien Noisser, Andreas Eigenberger, Maximilian Weiherer, Stephan Seitz, Lukas Prantl, Vanessa Brébant

**Affiliations:** 1 University Centre of Plastic, Aesthetic, Hand and Reconstructive Surgery, University Hospital Regensburg, Franz-Josef-Strauß-Allee 11, 93053 Regensburg, Germany; 2 Faculty of Mechanical Engineering, Ostbayerische Technische Hochschule Regensburg (OTH Regensburg), Regensburg, Germany; 3 Regensburg Medical Image Computing (ReMIC), Ostbayerische Technische Hochschule Regensburg (OTH Regensburg), Regensburg, Germany; 4 Department of Obstetrics and Gynecology, Caritas Hospital St. Josef, University of Regensburg, Regensburg, Germany

We would like to thank the authors for their great interest in our study and your engagement with our findings. In the following, we would like to respond to your questions and comments as best as possible.

Our study describes a very rare clinical picture of congenital breast asymmetry. These patients are very young at the time of corrective surgery and usually live with the post-operative result for most of their lives. The aim of our study was to identify objective criteria that have a positive effect on the subjective long-term satisfaction of this special group of patients. Several forms of congenital breast asymmetry were examined, to be able to generate as large a collective as possible, despite the rarity of the clinical picture. Although our study collective of 34 patients is not unusual compared to similar studies of congenital breast asymmetry (Kuzbari et al. [1] *n* = 30; Neto et al. [2] *n* = 35; Eder et al. [3] *n* = 28), the small collective should still be viewed critically, as it allows only limited generalizations [4].

It is interesting to note that in medicine, patients and surgeons may evaluate an outcome as favorable differently [5]. Our study aims to help identify this gap in the context of long-term outcomes in congenital breast asymmetry and consequently provide opportunities to close it. Our data were mainly gathered through manual measurements, patient-reported outcome measures (Breast Q™), and breast volumetry based on 3D scans (Vectra® H2, Canfield Scientific) [4].

We agree that it would be interesting to investigate the influence of mastopexy and MRP on outcome satisfaction. The patients in our study received either lipofilling or silicone implant therapy, which was applied unilaterally or bilaterally. Furthermore, a number of the patients in the collective underwent breast reduction or mastopexy additionally. A comparison between surgical techniques was not a question of the present study. We focused on post-operative visible or measurable criteria that influence patients’ subjective satisfaction. Nevertheless, we would like to thank you for the interesting angle, to connect a corresponding follow-up study on the influence of various surgical techniques on patient satisfaction.

We agree that SN-N is not irrelevant in our study either, but merely did not reveal any significant correlation in our collective. This result is quite remarkable and could be an indication of the aforementioned gap between the assessments of outcome reached by patients and physicians [5]. Other studies have also highlighted the NAC as a particular focus of outcome assessment in patients [6, 7], which underlines the results of our study. Patient and surgeon may have different foci [5]. However, to increase patient satisfaction, to which this study aims to contribute, one should consider the focus of the patient even more. Important consequences for surgery can therefore be derived from our work. We may conclude that a greater focus on the NAC by surgeons leads to greater patient satisfaction in our collective in the long term.

It is true that many factors can influence the long-term satisfaction of patients. We agree that limiting patient satisfaction to areola diameter or mean difference between left and right diameter would not be appropriate and was not the conclusion of our study. We would like to point out here that an average overall satisfaction of 74% was achieved, which is already a good result overall. This is in part the subject of a further publication. We would like to clarify that our study focuses on quality assurance and improvement and thus aims to contribute to elucidating which details or aspects of an already good result need even greater attention to get even closer to 100% subjective patient satisfaction.

Regarding our methodology, we would like to point out that we used one of the most innovative techniques currently available on the market, the 3D scanning system Vectra® H2 (Canfield Scientific, USA), which is frequently used in the current literature [8–12]. It was particularly interesting that the volume difference calculated by 3D technology had a significant influence on satisfaction with similarity of the breasts. Following this result of our study, we recommend investigating the use of this system in real time during surgery in further studies.

In addition, manual measurements were also recorded in our study. We explicitly point out that these also include the inferior mammary fold, differences in breast width and projection, as well as positioning of the nipple [4]. Although significant correlations were not found for all parameters in our study, we agree that they could exist and recommend analyzing the results in more detail in a multi-center study with a greater number of patients.

Thank you for the reference to the study on Scandinavian women by Sandberg et al. [13]. The results are interesting, but we would like to point out that Sandberg et al. took healthy women without surgery as a collective [13]. In our study, the research refers to patients with congenital breast asymmetry who were examined postoperatively. Because patients with congenital breast asymmetry are explicitly excluded, the results of Sandberg et al. [13] are not transferable to our study. However, it would be an interesting approach for a follow-up study to examine the transferability to our collective.

In summary, the small patient population, among others, must be seen as limitation, but our study still impresses with the use of the most innovative 3D scan technology and the analysis of the post-operative outcome with a focus on the long-term post-operative perspective. We are pleased to be able to contribute to improving long-term outcomes for patients with this rare condition. However, we recommend repeating these initial findings in a multi-center study with a larger population. We would like to thank you for your letter and hope that we have addressed the questions you raised.
